# Modified Target Delineation and Moderately Hypofractionated Radiotherapy for High-Grade Glioma: A Randomized Clinical Trial

**DOI:** 10.1001/jamanetworkopen.2025.23053

**Published:** 2025-07-24

**Authors:** Liangzhi Zhong, Pu Zhou, Lu Chen, Diangang Chen, Li Wen, Xinwei Diao, Anmei Zhang, Yixing Gao, Guangpeng Chen, Xueqin Li, Shaojiang Huang, Kai Niu, Yuchun Pei, Guolong Liu, Shengqing Lv, Guanghui Li

**Affiliations:** 1 Cancer Research Institute of the Chinese People’s Liberation Army, Xinqiao Hospital, Army Medical University, Chongqing, People’s Republic of China; 2 Department of Oncology, Shapingba Hospital affiliated to Chongqing University, Chongqing University, Chongqing, People’s Republic of China; 3 Department of Radiology, Xinqiao Hospital, Army Medical University, Chongqing, People’s Republic of China; 4 Department of Pathology, Xinqiao Hospital, Army Medical University, Chongqing, People’s Republic of China; 5 Department of Neurosurgery, Xinqiao Hospital, Army Medical University, Chongqing, People’s Republic of China

## Abstract

**Importance:**

Optimizing irradiation volumes and evaluating the effect of dose escalation on total and fractionated doses are critical for improving outcomes in high-grade glioma (HGG).

**Objective:**

To assess the efficacy of modified target delineation guided by multimodal magnetic resonance imaging and white matter tracts combined with moderately hypofractionated simultaneous boost intensity-modulated radiotherapy (HSIB-IMRT) in patients with newly diagnosed HGG.

**Design, Setting, and Participants:**

This single-center, 2-arm, open-label randomized clinical trial enrolled 154 patients aged 18 to 70 years with histologically confirmed, newly diagnosed HGG at a Chinese medical center from January 1, 2018, to August 31, 2022. Follow-up was completed in June 2024.

**Interventions:**

Patients were randomized to receive modified target delineation guided by multimodal magnetic resonance imaging and white matter tracts combined with HSIB-IMRT (experimental arm) or standard IMRT per guideline recommendations (standard arm). Both arms received concurrent and adjuvant temozolomide chemotherapy.

**Main Outcomes and Measures:**

The primary end point was progression-free survival (PFS). The secondary end point was overall survival (OS).

**Results:**

Among 154 enrolled patients (76 in the experimental arm and 78 in the standard arm; 85 [55.2%] male; median [range] age, 51.5 [23.0-70.0] years), the median (range) follow-up duration was 22 (4-76) months, with 96 deaths by June 2024. The median PFS was 15.5 months (95% CI, 11.7-19.3 months) in the experimental arm and 13.5 months (95% CI, 8.7-18.3 months) in the standard arm (*P* = .89). The median OS was 27.0 months (95% CI, 13.9-40.1 months) in the experimental arm and 21.0 months (95% CI, 18.0-24.0 months) in the standard arm (*P* = .24). The clinical target volume in the experimental arm (CTV1: median [range], 116.7 [20.2–370.7 cm^3^]; CTV2: median [range], 174.4 [34.5-463.2 cm^3^]) was significantly smaller than the clinical target volume in the standard arm (median [range], 225.0 [70.2-542.1 cm^3^]; *P* < .001). Recurrence rates within, outside, and multicentric to the target volume were comparable between arms. Grade 3 or 4 adverse events occurred in 4 patients (5.3%) in the experimental arm and 3 (3.8%) in the standard arm (*P* = .72).

**Conclusions and Relevance:**

In this randomized clinical trial, modified target delineation with HSIB-IMRT demonstrated comparable PFS and OS to standard IMRT in patients with newly diagnosed HGG, while significantly reducing the irradiation target volume without increasing the recurrence rates outside the target volume. These results suggest valuable insights for future research aimed at personalized, reduced volume strategies to optimize outcomes and minimize neurotoxicity in HGG.

**Trial Registration:**

ChiCTR.org.cn Identifier: ChiCTR1800014396

## Key Points

**Question:**

Does modified target delineation combined with moderately hypofractionated simultaneous boost intensity-modulated radiotherapy (HSIB-IMRT) improve the clinical outcomes for patients with newly diagnosed high-grade glioma (HGG)?

**Findings:**

In this randomized clinical trial of 154 patients with HGG, modified target delineation combined with moderately HSIB-IMRT did not improve progression-free or overall survival; however, it significantly reduced the irradiation target volume compared with standard IMRT. The experimental approach demonstrated equivalent efficacy to standard IMRT, without increasing the recurrence outside the target volume.

**Meaning:**

These results suggest that modified target delineation combined with HSIB-IMRT provides clinical efficacy comparable to standard IMRT for newly diagnosed HGG compared with the potential for reduced irradiation volumes.

## Teaser

This randomized clinical trial of patients with high-grade glioma compares the efficacy and safety of multimodal magnetic resonance imaging and white matter tract–guided target delineation combined with hypofractionated simultaneous boost intensity-modulated radiotherapy vs standard intensity-modulated radiotherapy.

## Introduction

High-grade gliomas (HGGs), particularly glioblastoma, are characterized by aggressive progression and poor prognosis, emphasizing the need to investigate better treatment strategies.^1,2^ Notably, HGGs pose a unique neurocognitive burden: more than 50% of patients with brain or central nervous system tumors develop moderate-to-severe cognitive impairment—the highest rate observed across all cancer types. This dual challenge of limited survival and high neurotoxicity risk highlights the urgent need for therapeutic strategies that achieve both effective tumor control and cognitive preservation, a patient-centered goal often undermined by conventional treatment intensification.^3^

According to the 2016 Radiotherapy and Oncology Group (RTOG)/NRG Guidelines, the clinical target volume (CTV) of HGGs, which is considered a high-risk factor for subclinical and microscopic glioma cell spread, is defined as the volume expanded 2 cm from the resection cavity plus cytotoxic edema.^1^ This approach has remained relatively unchanged during the past 2 decades despite advances in precision radiotherapy.^2^ Brain white matter tracts, composed of myelinated nerve fibers, serve as pathways for glioma cell migration. Since Scherer’s seminal work,^4^ it has been established that glioma cells preferentially spread along white matter tracts rather than infiltrating uniformly in all directions.^5,6,7^ These tracts facilitate long-range migration of glioma cells to distant brain regions.^4,8^ Conventional CTV expansion techniques do not account for the anisotropic nature of glioma cell infiltration, often resulting in excessive irradiation of normal brain tissue. Multimodal magnetic resonance imaging (MRI), including diffusion tensor imaging and T2-weighted and fluid-attenuated inversion recovery (FLAIR) sequences, provides valuable information on glioma cell infiltration pathways and cytotoxic edema. Therefore, refining CTV delineation along the white matter tracts using multimodal MRI may reduce irradiation volumes in patients with HGG.

Although most total dose–escalated radiotherapy regimens showed no significant survival benefit over conventional fractionation (60 Gy in 30 fractions) in glioblastoma, preliminary evidence suggests that moderately hypofractionated simultaneous boost intensity-modulated radiotherapy (HSIB-IMRT) exhibits favorable tolerability in this patient population.^9^ In a retrospective analysis^10^ of 80 patients with newly diagnosed glioblastoma treated with HSIB-IMRT at our institution, the median overall survival (OS) was 21 months, the median progression-free survival (PFS) was 15 months, and the 5-year OS rate was 13.4%. Building on these findings, we conducted a single-center randomized clinical trial to compare the efficacy and safety of multimodal MRI and white matter tract–guided target delineation combined with HSIB-IMRT vs RTOG-recommended standard IMRT in patients with newly diagnosed HGG.^2^

## Methods

### Study Design 

This single-center, 2-arm, intention-to-treat, open-label randomized clinical trial (see the trial protocol in Supplement 1) was approved by the Ethics Committee of the Army Medical University and registered in the Chinese Clinical Trial Registration Center. All participants provided written informed consent. The study followed the Consolidated Standards of Reporting Trials (CONSORT) reporting guideline.

### Eligibility Criteria and Randomization

**Figure 1.**
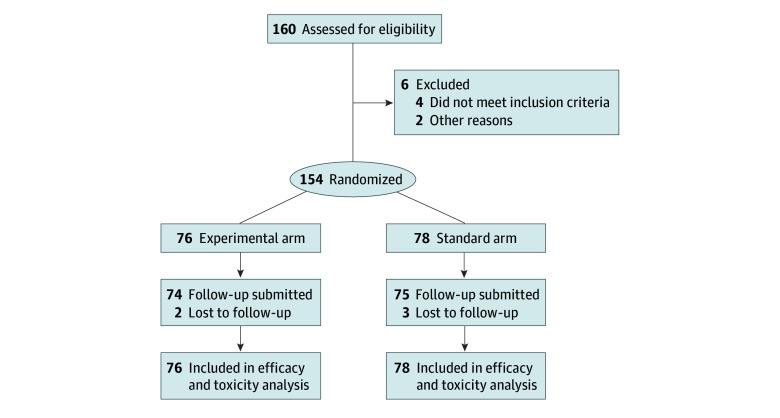
Study Flow Diagram

Patients who met the following criteria were enrolled at a Chinese medical center from January 1, 2018, to August 31, 2022: (1) pathologically confirmed HGG per the 2016 World Health Organization classification of the central nervous system tumors after surgical resection or biopsy^11^; (2) expected survival time of 3.0 months or longer; and (3) age of 18 to 70 years (Figure 1). Of the 160 patients evaluated for eligibility, 6 were excluded. Patients were randomized in a 1:1 ratio to experimental or standard arm using computer-generated random numbers. An independent statistician, uninvolved in recruitment or outcome assessment, prepared the randomization list. Allocation concealment was maintained using opaque, sealed envelopes opened only after baseline assessments, ensuring investigators remained blinded to group assignments until randomization. Participants' race and ethnicity were not specifically collected or reported in this study because the study population was highly homogeneous, consisting exclusively of individuals recruited from a single geographic region within China. Within the context of this specific clinical investigation and its objectives, race and ethnicity were not considered relevant variables or potential confounders for the outcomes being assessed.

### Computed Tomography Simulation Positioning, Scanning, and Image Registration

All patients underwent preoperative and postoperative brain multimodal MRI, including T1-weighted, T2-weighted, T2-weighted and FLAIR or apparent diffusion coefficient, diffusion tensor imaging, diffusion-weighted imaging, and 3-dimensional magnetic resonance spectroscopy. Between 2 and 6 weeks after surgery, patients were positioned supinely with their heads secured in a customized immobilization and underwent computed tomography (CT) simulation. CT images were then transferred to the treatment planning system (Varian Medical Systems). Multimodal MRIs were integrated into the planning system and registered to the simulation CT images for treatment planning.

### Target Volume Definition and Irradiation Dosage Prescription

In the experimental arm, target volumes were delineated using multimodal MRIs and white matter tract anatomical atlas.^12^ Gross tumor volume (GTV) comprised the resection cavity plus residual T1 contrast-enhancing tumor (if present). The CTV was defined as (1) CTV1, including GTV and cytotoxic edema identified on preoperative and postoperative multimodal MRI, and (2) CTV2, expanding 1 cm from CTV1 along white matter tracts, excluding adjacent brain gyrus with normal MRI signals (eMethods in Supplement 2). Margins were modified to avoid organs at risk. Planning target volumes (PTVs) were generated expanding 3 mm from GTV, CTV1, and CTV2 to create PGTV, PCTV1, and PCTV2, respectively. The prescribed HSIB-IMRT doses were 64 to 66 Gy to PGTV, 60 to PCTV1, and 54 Gy to PCTV2 in 27 fractions (once daily 5 days per week).

**Figure 2.**
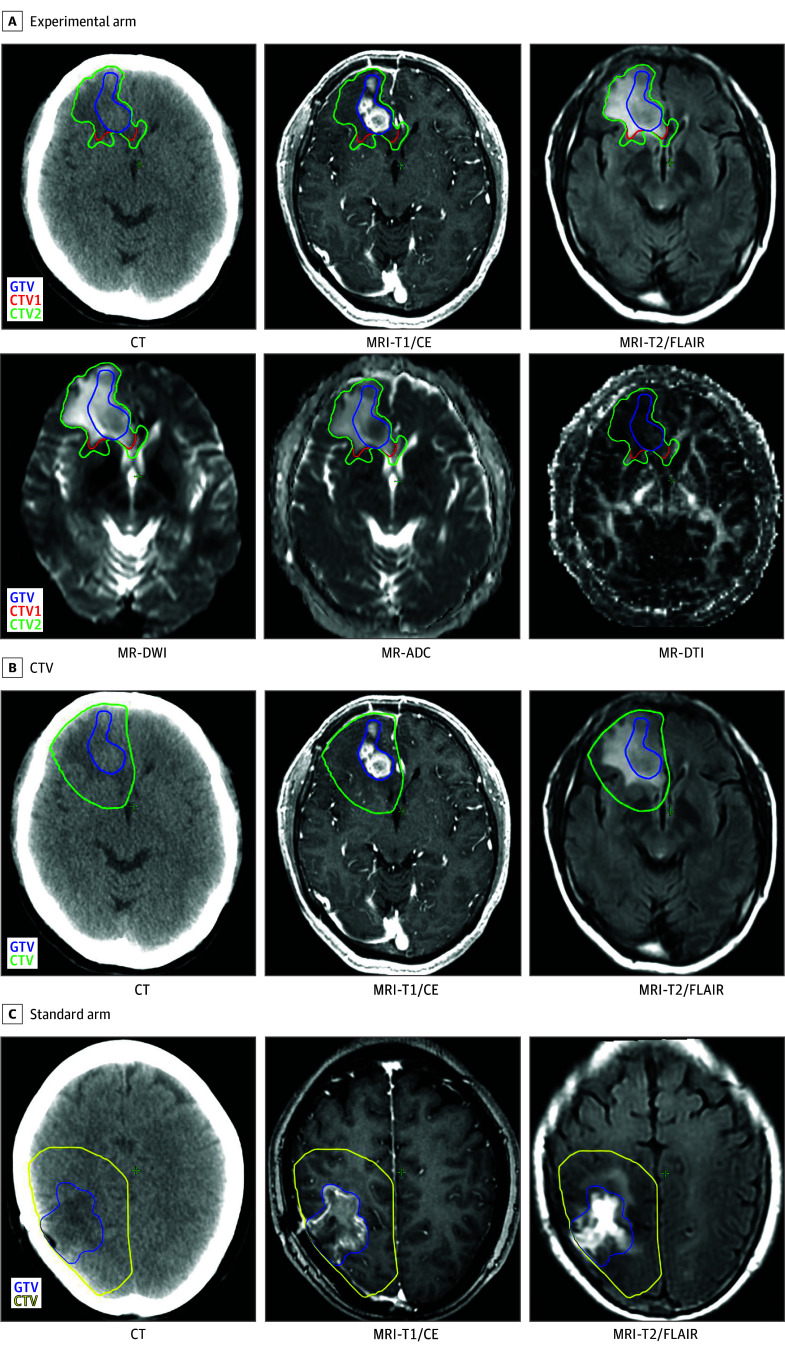
Target Volume Definition A case of right frontal glioblastoma (experimental arm) (A) with the clinical target volume (CTV) (yellow contour) delineated according to the approach of a case or right occipitoparietal glioblastoma (standard arm) (C). ADC indicates apparent diffusion coefficient; CT, computed tomography; DTI, diffusion tensor imaging; DWI, diffusion-weighted imaging; GTV, gross tumor volume; MR, magnetic resonance; MRI, magnetic resonance imaging; T1/CE, T1 weighted and contrast enhanced; T2/FLAIR, T2 weighted and fluid-attenuated inversion recovery.

In the standard arm, the GTV was defined similarly, with the CTV comprising the GTV plus a 2-cm margin and cytotoxic edema area, per RTOG recommendations.^2^ The PGTV and PCTV were obtained by expanding 3 mm from the GTV and CTV, respectively, with margin adjustment for organs at risk. Standard IMRT delivered 50 Gy in 25 fractions to PCTV in the first phase and followed by 10 Gy in 5 fractions to PGTV in the second phase (2 Gy/d 5 days/week). Target volumes for both arms were finalized by a multidisciplinary glioma team (Figure 2).

### Concurrent and Adjuvant Chemotherapy

Patients in both arms received concurrent temozolomide chemotherapy (75 mg/m^2^ daily) during radiotherapy and 6 cycles of adjuvant temozolomide chemotherapy (150-200 mg/m^2^/d for 5 days every 4 weeks).^13^ The total number of temozolomide cycles was determined according to the patients’ general condition, adherence, economic situation, and disease progression.

### Follow-Up, Efficacy Evaluation, and Adverse Event Monitoring

Patients were monitored weekly during radiotherapy through medical record reviews, physical examinations, and hematologic tests, followed by monthly assessment after radiotherapy. MRI assessments were conducted at 1 and 3 months after radiotherapy and every 3 months thereafter. When tumor progression or necrosis could not be confirmed by routine MRI, additional imaging (eg, multimodal MRI and positron emission tomography plus CT) was conducted. Follow-up was completed in June 2024. Treatment response was assessed according to the Response Assessment in Neuro-Oncology (RANO) criteria.^4^ Tumor recurrence or progression was confirmed by multidisciplinary team discussion or pathological examinations after subsequent operation. Recurrence patterns were categorized into 3 types: (1) within the target volume (CTV2 for the experimental arm and CTV for the standard arm), (2) outside the target volume, or (3) multicentric (defined as simultaneous recurrence inside and outside the target volume or within and outside the brain parenchyma). Adverse events were monitored via routine hematologic tests, symptoms inquiries, and medical examinations, graded according to RTOG and the European Organisation for Research and Treatment of Cancer (EORTC) criteria for radiation therapy toxicity.^14,15^

### Statistical Analysis

The primary end point was PFS, and the secondary end point was overall survival, measured from the date of surgery to progression or death, respectively, or censored at the last follow-up. On the basis of the Stupp regimen’s median PFS of 6.9 months and our retrospective study’s median PFS of 15 months,^10,13^ we assumed a hazard ratio (HR) of 0.5. Referencing previous reports,^16,17^ we estimated a median PFS of 10 months for patients with HGG treated with the Stupp regimen. With a 24-month accrual period, 36-month maximum follow-up, and 5% dropout rate, 152 patients (76 per arm) were required to achieve 80% power and a 2-sided type I error of 0.05, expecting 66 events for the primary analysis of PFS.

Qualitative variables were presented as numbers (percentages) and continuous variables as medians (ranges). Differences between arms were evaluated using the χ^2^ or Fisher exact test for qualitative variables and the Mann-Whitney *U* test for continuous variables. Survival outcomes were calculated with the Kaplan-Meier method, with differences compared via the log-rank test. Cox proportional hazards regression was performed to examine the effect of different survival factors. The proportional hazards assumption was verified; variables meeting the assumption used Cox proportional hazards regression, whereas for those violating the assumption the HRs were interpreted as weighted means during the entire follow-up period. Univariate analysis identified variables with *P* < .10 for inclusion in the multivariate model. Statistical significance was defined as 2-tailed *P* < .05. Analyses were performed using SPSS version 29.0 (IBM Corp).

## Results

### Patient Enrollment and Treatment

Among 154 enrolled patients (85 [55.2%] male and 69 [44.8%]; median [range] age, 51.5 [23.0-70.0] years), 76 patients (51 with glioblastoma and 25 with grade III glioma) were randomized to the experimental arm and 78 (57 with glioblastoma and 21 with grade III glioma) to the standard arm (Figure 1). Baseline characteristics and demographics were balanced (Table 1). *MGMT* (OMIM 156569) methylation and *TERT* (OMIM 187270), *ATRX* (OMIM 300032), and *BRAF* (OMIM 164757) gene variants were tested in 74 patients. Additionally, 56 patients (28 per arm) with recurrence underwent second-line treatment.

**Table 1.** Demographic and Baseline Clinical Characteristics of the Study Patients

Characteristic	Patients, No. (%)	
	Experimental arm (n = 76)	Standard arm (n = 78)
Sex		
Male	43 (56.6)	42 (53.8)
Female	33 (43.4)	36 (46.2)
Age, y		
Median (range)	53 (25-69)	51 (23-70)
<55	40 (52.6)	46 (59.0)
≥55	36 (47.4)	32 (41.0)
WHO grade		
III	25 (32.9)	21 (26.9)
IV	51 (67.1)	57 (73.1)
Extent of surgery		
Complete resection	69 (90.8)	64 (82.1)
Partial resection	6 (7.9)	12 (15.4)
Biopsy	1 (1.3)	2 (2.6)
Time to beginning radiotherapy, d		
Median (range)	38.5 (23-69)	38.5 (16-127)
≤42	51 (67.1)	52 (66.7)
>42	25 (32.9)	26 (33.3)
Adjuvant temozolomide cycles		
Median (range)	6 (0-20)	6 (0-35)
<6	24 (31.6)	31 (39.7)
6	29 (38.2)	19 (24.4)
>6	23 (30.2)	28 (35.9)
*MGMT* status		
Methylated	23 (30.3)	16 (20.5)
Unmethylated	22 (28.9)	13 (16.7)
Unknown	31 (40.8)	49 (62.8)
*IDH*		
Variant	20 (26.3)	26 (33.3)
Wild type	51 (67.1)	49 (62.8)
Unknown	5 (6.6)	3 (3.8)
1p19q		
Codeleted	8 (10.5)	5 (6.4)
Noncodeleted	37 (48.7)	24 (30.8)
Unknown	31 (40.8)	49 (62.8)
*TERT* C228T		
Variant	22 (28.9)	16 (20.5)
Wild type	23 (30.3)	13 (16.7)
Unknown	31 (40.8)	49 (62.8)
*TERT* C250T		
Variant	10 (13.2)	6 (7.7)
Wild type	31 (40.8)	22 (28.2)
Unknown	35 (46.1)	50 (64.1)
*BRAF*-V600E		
Variant	0	0
Wild type	42 (55.3)	28 (35.9)
Unknown	34 (44.7)	50 (64.1)

Abbreviation: WHO, World Health Organization.

### PFS and OS

**Figure 3.**
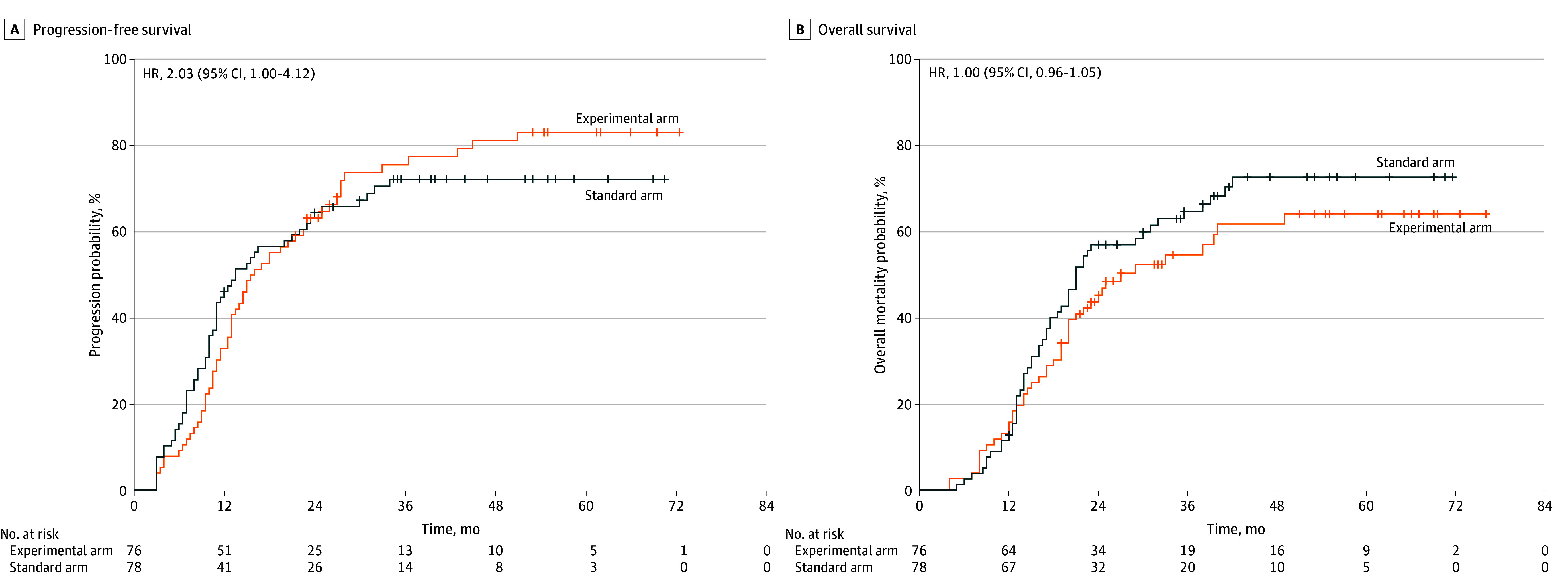
Progression-Free Survival and Overall Survival HR indicates hazard ratio.

With a median follow-up of 22 months (range, 4-76 months), 96 deaths occurred by June 2024. The median PFS was 15.5 months (95% CI, 11.7-19.3 months) in the experimental arm and 13.5 months (95% CI, 8.7-18.3 months) in the standard arm (*P* = .89). The median OS was 27.0 months (95% CI, 13.9-40.1 months) in the experimental arm and 21.0 months (95% CI, 18.0-24.0 months) in the standard arm (*P* = .24) (Figure 3).

Univariate analysis identified age, *MGMT* promoter methylation status, *IDH* (OMIM 147700) variant status, adjuvant chemotherapy cycles, and pathological grade as factors associated with PFS and OS. Multivariate analysis confirmed age, *IDH* variant status, and adjuvant chemotherapy cycle as factors associated with PFS and pathological grade, *IDH* variant status, and adjuvant chemotherapy cycles as factors associated with OS (eTable 1 in Supplement 2).

### Target Volume Comparison

The median (range) GTV volume was similar between the experimental arm (46.8 [1.2–224.8] cm^3^) and standard arm (49.4 [3.8-275.7] cm^3^) (*P* = .62). However, CTV1 (median [range], 116.7 [20.2–370.7] cm^3^) and CTV2 (median [range], 174.4 [34.5-463.2] cm^3^) in the experimental arm were significantly smaller than the CTV (median [range], 225.0 [70.2–542.1] cm^3^) in the standard arm (*P* < .001). When the CTV of patients in the experimental arm was delineated with the standard arm delineation approaches, CTV2 was also significantly smaller than the CTV (median [range], 239.4 [98.2-505.6] cm^3^; *P* < .001).

### Recurrence Patterns

Disease progression occurred in 114 patients (59 [77.6%] in the experimental arm and 55 [70.5%] in the standard arm). Imaging-confirmed recurrence was observed in 79 patients (38 experimental and 41 standard). In the experimental arm, 24 recurrences (63.2%) were within CTV2 compared with 21 (51.2%) within CTV in the standard arm. Recurrences outside the target volume occurred in 8 (21.1%) and 15 (36.6%) patients in the experimental and standard arms, respectively (*P* = .18). Multicentric recurrences were observed in 13 (34.2%) and 7 (17.1%) paitnets in the experimental and standard arms, respectively (*P* = .16). According to the RANO criteria, 14 and 12 patients in the experimental and standard arms, respectively, were classified as unknown without confirmation by MRI or further surgery.

The distant recurrence rates were 4.4% (1 of 23 patients) and 31.8% (7 of 22 patients) in patients with and without *MGMT* promoter methylation in the experimental arm, respectively, compared with 25.0% (4 of 16 patients) and 23.1% (3 of 13 patients) in the standard arm, respectively. For *IDH* status, distant occurrence was observed in 2 of 51 patients (3.9%) with *IDH* wild-type variants and 10 of 20 patients (50.0%) with *IDH* variants in the experimental arm compared with 9 of 49 (18.4%) and 7 of 26 (26.9%) in the standard arm, respectively (eTable 2 in Supplement 2).

### Adverse Events

During concurrent and adjuvant chemotherapy, the most prevalent acute adverse reactions were dizziness, headache, nausea, vomiting, loss of appetite, and hematologic toxicicity (mostly grades 1 and 2). Anemia, neutropenia, and thrombocytopenia were observed in 43 (56.6%), 19 (25.0%), and 15 (19.7%) of the 76 patients in the experimental arm and 46 (59.0%), 13 (16.7%), and 14 (17.9%) of the 78 patients in the standard arm, respectively. Grade 3 to 4 adverse events were reported in 4 (5.3%) and 3 (3.8%) patients in the experimental and standard arm, respectively, without statistical significance (*P* = .72) (Table 2).

**Table 2.** Adverse Events in Patients in the Experimental and Standard Arms

Adverse event	Patients, No. (%)		*P* value
	Experimental arm (n = 76)	Standard arm (n = 78)	
Neutropenia			
Grades 1-2	18 (23.7)	12 (15.4)	.43
Grades 3-4	1 (1.3)	1 (1.3)	
Anemia			
Grades 1-2	42 (55.3)	44 (56.4)	.83
Grades 3-4	1 (1.3)	2 (2.6)	
Thrombocytopenia			
Grades 1-2	13 (17.1)	14 (17.9)	.35
Grades 3-4	2 (2.6)	0	
Fatigue			
Grades 1-2	2 (2.6)	1 (1.3)	.55
Grades 3-4	0	0	
Nausea			
Grades 1-2	28 (36.8)	33 (42.3)	.49
Grades 3-4	0	0	
Vomiting			
Grades 1-2	5 (5.3)	9 (11.5)	.28
Grades 3-4	0	0	
Headache			
Grades 1-2	9 (11.8)	13 (16.7)	.39
Grades 3-4	0	0	
Hypersomnia			
Grades 1-2	0	1 (1.3)	.33
Grades 3-4	0	0	

All patients underwent a Mini-Mental State Examination (MMSE) 1 week before radiotherapy, with median score of 28 points in both the experimental arm (range, 24-30) and standard arm (range, 23-30). One week after completing adjuvant chemotherapy, 103 patients were reassessed. The median MMSE scores were 28 points for the experimental arm and 27 points for the standard arm, respectively.

## Discussion

The Stupp regimen,^13^ combining radiotherapy with concurrent and adjuvant temozolomide chemotherapy, remains the standard treatment for newly diagnosed glioblastoma. However, outcomes for glioblastoma with multidisciplinary treatment remain suboptimal.^18,19,20^ Advanced radiotherapy techniques enable precise dose delivery while sparing organs at risk, yet late neurocognitive dysfunction resulted from normal brain tissue irradiation persists as a concern.^21^ Optimal total radiotherapy dose, fractionation, and target volume for HGG radiotherapy require further investigation to improve efficacy and minimize toxicity. Unlike prior studies,^1,2,9^ this trial escalated total and fractionated doses while reducing target volume in patients with HGG.

Ongoing research investigates whether higher total or fractionated radiation dose improve survival in patients with HGG.^22,23^ A prior retrospective analysis^10^ suggested that HSIB-IMRT improved outcomes in glioblastoma compared with other literature. However, this trial found that HSIB-IMRT did not significantly prolong the median PFS and median OS and did not reduce the recurrence within target volume in patients with HGG compared with standard IMRT. These results suggest that increasing the total or fractionated doses does not improve tumor control in HGG. Recently, Laprie et al^24^ reported that dose-escalated radiotherapy (72 Gy) targeting magnetic resonance spectroscopy imaging–defined metabolic abnormalities did not improve OS in newly diagnosed glioblastoma. Other studies^25,26,27^ have also shown that higher radiation dosages do not extend OS or improve tumor control in newly diagnosed HGG. Combining radiotherapy with other treatment strategies, such as targeted drugs or radiosensitizers, may be a better option to improve antitumor efficacy in HGG. For example, adjuvant temozolomide with interferon alfa treatment prolongs the survival time of patients with HGG.^28^

In this trial, HSIB-IMRT was safe for patients with HGG, with no significant differences in toxic effects between arms. The most common adverse events were hematologic toxic effects and gastrointestinal effects associated with chemotherapy and radiotherapy, consistent with previous reports.^13,29,30^

There is no consensus on optimal irradiation target volumes for HGG across radiotherapy centers. The RTOG-9503 and EORTC-26981 trials suggested a 2- to 3-cm margin around GTV, with or without inclusion of cytotoxic edema assessed by T2-weighted and FLAIR sequences.^2,11^ These suggestions do not account for the anisotropic infiltration patterns of glioma cells, resulting in larger irradiation volumes. Accurate target definition is vitally crucial for reducing normal brain tissue irradiation and preserving neurologic function. A panel of radiation oncologists specializing in central nervous system diseases emphasized the need for further research to reduce target volume based on glioblastoma’s infiltrative nature.^3^ Irradiation of normal brain tissue near the GTV is still worthy of further investigation.

Previous studies^31,32^ have confirmed glioma cell infiltration in edematous brain tissue. Abnormal edema caused by subdiagnostic tumor infiltration serves as an early indicator of glioma progression as incorporated in the RANO criteria.^2,8,33^ Multimodal MRI, including T2-weighted and FLAIR sequences, diffusion-weighted imaging, apparent diffusion coefficient and 3-dimensions MRS, combined with the white matter tract atlas, provides accurate information on glioma infiltration pathway.^33,34,35,36,37^ In this study, CTV1 and CTV2 delineation, guided by these multimodal MRI data and white matter tracts, reduced irradiation volume by approximately 22.5% in the experimental arm. This reduction did not compromise radiotherapy efficacy or alter recurrence patterns compared with the standard arm. Given that radiation-induced neurotoxicity correlates with the radiation dose exposed to normal brain tissue,^38^ the modified target volume significantly reduced the radiation dose to adjacent brain gyri near the GTV, likely enhancing neuroprotection.

Notably, the experimental arm showed a lower incidence of distant recurrence in patients with HGG with *MGMT* promoter methylation compared with the standard arm. Previous studies^39,40,41^ have reported that *MGMT* promoter methylation is associated with higher rates of distant glioma recurrence. Brandes et al^39^ and Minniti et al^40^ reported that *MGMT*-methylated gliomas had increased distant relapses compared with unmethylated cases. Our findings indicated that HSIB-IMRT may reduce distant relapse in patients with *MGMT*-methylated HGG. This finding aligns with the results of the study by Laack et al,^41^ which suggests that dose escalation may particularly benefit patients with *MGMT*-methylated HGG. Precise local intensification strategies may effectively mitigate this localized failure in this subgroup.

### Limitations

This study has limitations. The sample size calculation assumed an HR of 0.5 based on promising outcomes from previous retrospective studies,^10,16,17^ potentially overestimating the treatment effect. In addition, the observed HR of 0.87 suggests the trial was underpowered to detect smaller but clinically meaningful differences, necessitating future trials to consider more conservative effect size estimates or adaptive designs for such uncertainties. Suboptimal treatment adherence, with few patients with recurrent disease receiving second-line therapy and one-third completing fewer than 6 chemotherapy cycles, could compromise HGG survival, whereas simultaneous alteration of both radiation dose and target volume may obscure individual parameter contributions to outcomes, complicating benefit-risk assessments. Furthermore, the MMSE’s limited sensitivity in detecting subtle neurocognitive impairments as a simplistic tool may obscure potential benefits of reduced radiation target volumes.

## Conclusions

This randomized clinical trial demonstrated that HSIB-IMRT had similar PFS and OS to conventional IMRT in patients with newly diagnosed HGG. The modified CTV delineation reduced the target volumes without compromising efficacy or altering recurrence patterns. This study provides valuable insights for future research aimed at personalized, reduced volume strategies to optimize outcomes and minimize neurotoxicity in HGG.

## Notes

Supplement 1.
Trial Protocol

Supplement 2. **eMethods.** Target Volume Definition in Detail **eTable 1.** Univariate and Multivariate Analysis for Progression-Free Survival and Overall Survival **eTable 2.** Patterns of Recurrence

Supplement 3.
Data Sharing Statement
