# Errors in Figure 3 and Visual Abstract Findings

**DOI:** 10.1001/jamanetworkopen.2025.34026

**Published:** 2025-08-27

In the Original Investigation titled “Modified Target Delineation and Moderately Hypofractionated Radiotherapy for High-Grade Glioma: A Randomized Clinical Trial,”^1^ published July 24, 2025, there were errors in Figure 3 and in the visual abstract. In Figure 3, the y-axis to panel A given as “Progression-free survival, %” should have been “Progression probability, %” and the y-axis in panel B given as “Overall survival, %” should have been “Overall mortality probability, %.” In the visual abstract Findings, the y-axis given as “Progression-free survival, %” should have been “Progression probability, %.” The legend below the image presented as “Progression-free survival” should have been “Modified target delineation and radiotherapy for high-grade glioma.” This article has been corrected.^1^
