# Supplementary material for: Modified Target Delineation and Moderately Hypofractionated Radiotherapy for High-Grade Glioma: A Randomized Clinical Trial
Source: JAMA Netw Open. 2025 Jul 24;8(7):e2523053. doi: 10.1001/jamanetworkopen.2025.23053 (PMC12290737; doi:10.1001/jamanetworkopen.2025.23053)
Supplement: Supplement 1. — Trial Protocol [file jamanetwopen-e2523053-s001.pdf]

Trial Protocol

Clinical study of hypofractionated simultaneous integrated boost intensity-modulated radiotherapy for postoperative high-grade glioma guided by 3D-MRS combined with neuroanatomy

Version number: V1.0

Version dated: 17 August 2017

Study department: Department of Oncology

Principal investigator: Liangzhi Zhong

1. Research background and significance

1.1. Summary

Glioma refers to tumors originating from glial cells and is the most common primary intracranial tumor. The WHO classification is grades I to IV, grades I and II are low-grade glioma (Low grade glioma, LGG), and grades III and IV are high-grade glioma (High grade glioma, HGG). The treatment of glioma is mainly surgical resection of tumors, combined with radiotherapy, chemotherapy and other comprehensive treatment methods.

Postoperative radiotherapy can kill and inhibit residual tumor cells, prolong the recurrence time and survival of patients, and divide external radiotherapy is the standard therapy for glioma. With the continuous development and progress of precision radiotherapy technology, it has created a new clinical understanding of postoperative radiotherapy and become the mainstream of current radiotherapy technology. Glioma has the characteristics of recurrence and within the range of high recurrence risk, how to select radiotherapy technology and optimize local radiotherapy is one of the difficulties.

1.2. Radiotherapy of HGG

The comprehensive treatment of maximum safe resection surgery + postoperative concurrent chemoradiotherapy and adjuvant TMZ chemotherapy is the recommended treatment regimen by various major guidelines. The definition and dose of the target area of postoperative radiotherapy recommended by the current guidelines are: postoperative lesions and T2 / FLAIR abnormal signal area are GTV, CTV is obtained by 1 to 2 cm, PTV is formed by CTV amplification 0.3~0.5cm, CTV dose is 40-50 Gy, and GTV dose is 60 Gy / 30F.

In recent years, many scholars have done a lot of research in radiotherapy segmentation, including super segmentation and accelerate the segmentation of radiotherapy, radiotherapy, during the same time local segmentation of radiotherapy, radiotherapy, etc., reported different results, overall results, always improve the total dose of radiation technology failed to show obvious survival benefit, and change the segmentation pattern during the same period of radiation technology in the protection

of surrounding normal tissue at the same time further improve the tumor dose, show a survival advantage, even is changing the patients with malignant glioma recurrence pattern.

Based on the above research results and combined with the conditions of our unit, the radiotherapy scheme of this study is low segmentation intensity radiotherapy. The target area definition and dose given regimen are detailed in " V. Study method and Diagnostic criteria 5.1 Target area definition and dose setting ".

1.3. 3D-MRS in glioma radiotherapy

3D-MRS and intensity-modulated radiotherapy, mature technology and widely used, 3D-MRS for the differentiation of recurrence and granulation tissue after glioma treatment. Early we in glioma postoperative IMRT has done a lot of work, found that glioma with the corresponding nerve bundle as the main recurrence metastasis, therefore, we design this scheme, rely on 3D-MRS and neuroanatomical information, guide glioma strong target outline, expected to improve the residual tumor / high-risk recurrence area of radiation dose, increase the corresponding nerve bundle prevention dose, reduce the dose of normal brain tissue, so as to improve tumor control rate along the corresponding nerve recurrence risk, reduce the normal brain tissue damage, improve the quality of life of patients.

References

1. Compilation group of Guidelines for the Diagnosis and Treatment of Central Nervous System Glioma in China. Guidelines for the diagnosis and treatment of CNS glioma in China (2015) [J]. Chinese Journal of Medicine, 2016,96 (7): 485-509.

2. Badiyan SN,Markovina S,Simpson JR,et al.Radiation therapy dose escalation for glioblastoma multiforme in the era of temozolomide ［J］.Int J Radiat Oncol Biol Phys,2014,90(4):877-885.

3. Reddy K, Damek D, Gaspar LE, et al. Phase II trial of hypofractionated IMRT with temozolomide for patients with newly diagnosed glioblastoma multiforme. Int J Radiat Oncol Biol Phys, 2012 Nov 1; 84(3):655-60.

4. Lang Jinyi. Current status and prospect of clinical treatment of high-grade glioma

[J]. Chinese Journal of Cancer Prevention and Treatment, 2014,6 (4): 323-326.
5. Louis DN,Ohgaki H,Wiestler OD,et al.The 2007 WHO Classification of Tumours of the Central Nervous System ［J］.Acta Neuropathol,2007.114(2):97-109.
6. Stupp R,Hegi M,Jaeckle KA,et al.RTOG 0525:A Randomized Phase III Trial Comparing Standard Adjuvant Temozolomide(TMZ) With a Dose-Dense (dd) Schedule in Newly Diagnosed Glioblastoma(GBM) ［J］.J Clin Oncol ,2011,29(15_suppl):2006.
7. Hart MG,Garside R,Rogers G,et al,Temozolomide for high-grade glioma ［J］.Cochrane Database Syst Rev,2013,4(4):CD007415.
8. Bebawy JF,Perioperative steroid for peritumoral intracranial edema:A review of mechanisms,efficacy,and side effects ［J］.J Neurosurg Anesthesiol， 2012， 24(3):173-277.
9. Expert group of the Chinese Anti-Epilepsy Association. Expert consensus on the application of antiepileptic drugs after surgery for cranial diseases (trial) [J]. Chinese Journal of Neurosurgery, 2012.28 (7): 751-754.
10. Wen PY,Macdonald DR,Reardon DA,et al.Updated response assessment criteria for high-grade gliomas:response assessment in neuro-oncology working group ［J］.J Clin Oncol,2010,28(11):1963-1972.
11. Gu Mzhi, Yin Weibo, Yu Zihao, etc. Radiotherapy in Oncology (Fourth Edition) [M]. Beijing: China Union Medical College University Press, 2007:1350-1352.
12. Nabors LB,Portnow J,Ammiriati M,et al.CNS Cancers,version 1,2015 ［J］.J Nat Compr Canc Netw,2015,13(10):1191-1202.
13. Mao Ying. Glioma Mao Ying 2016 view [M]. Beijing: Science and Technology Academic Press, 2016:001-016.
14. Zhou B,Liu W.Post-traumatic glioma:report of one case and review of the literature[J].Int J Med Sci.2010,7(5):248-250.
15. Iuchi T,Hatano K,Narita Y,et al.Hypofractionated high-dose irradiation for the

treatment of maligant astrocytomas using simultaneous integrated boost technique by IMRT[J].Int J Radiat Oncol Biol Phys,2006,64(5):1317-1324.

16. Stupp R, Hegi ME, Mason WP, et al. Effects of radiotherapy with concomitant and adjuvant temozolomide versus radiotherapy alone on survival in glioblastoma in a randomised phase III study: 5-year analysis of the EORTC-NCIC trial. Lancet Oncol. 2009 May; 10(5):459-66.

Ma Wenbin, Li Yongning, Wang Renzhi. Review and interpretation of important node events in the comprehensive diagnosis and treatment of glioma in 40 years [J / CD]. Chinese Journal of Clinicians: Electronic edition, 2013,7 (14): 6217-6221.

18. National Comprehensive Cancer Network. NCCN guidelines: central nervous system cancers 2012 [Internet]. Fort Washington, PA: National Comprehensive Cancer Network; 2012 [cited 2014 Dec 1]. Available from: http://www.nccn.org/professionals/physician gls/f/guidelines.asp.

19. Darlix A, Baumann C, Lorgis V, et al. Prolonged administration of adjuvant temozolomide improves survival in adult patients with glioblastoma. Anticancer Res. 2013 Aug;33(8):3467-74.

20. Gai Xiaohui, Xue Xiaoying. Status and progress of radiotherapy dose segmentation in high-grade glioma [J]. Cancer prevention and Treatment Research, 2016,43 (3): 234-237.

21. Manickam M, Patrick P, Brian J,　et al.3D-MR Spectroscopic Imaging at 3Tesla for Early Response Assessment of Glioblastoma Patients during External Beam Radiation Therapy.Int J Radiat Oncol Biol Phys.2014 September 1; 90(1): 181–189.

22. LAWRENCE L. WALD, PH.D., SARAH J. et al.Serial proton magnetic resonance spectroscopy imaging of glioblastoma multiforme after brachytherapy，J Neurosurg 1997，87:525–534.

23. Ken, Laure V, Xavier F,et al.Integration method of 3D MR spectroscopy into treatment planning system for glioblastoma IMRT dose painting with integrated simultaneous boost,Radiation Oncology ,2013, 8:1.

24. Berkay Kanberoglu,Nina Z. David F,et al.Neuronavigation Using Three-Dimensional Proton Magnetic Resonance Spectroscopy Data,Stereotact Funct Neurosurg 2014;92:306–314.

25. QingShi Zeng , HePeng Liu, Kai Zhang,Noninvasive evaluation of cerebral glioma grade by using multivoxel 3D proton MR spectroscopy,Magnetic Resonance Imaging 29 (2011) 25-31.

2. Research objectives

2.1., Main indicators

1. Disease progression-free survival (Progression-Free-Survival, PFS): median PFS and disease progression-free survival rate.

2. Overall survival (Overall survival, OS): median OS and overall survival.

2.2., Secondary purpose

1. Explore the recurrence pattern (site) of the relapsed subjects, and further optimize the target area delineation and dose giving.

2. Evaluation of subject quality of life and neurocognitive function: EORTC QLQ-C30 (V3.0), Chinese version, MMSE.

3. Subject investigated

(I) Inclusion criteria:

All enrolled subjects must meet the following criteria:

1. Inclusion criteria related to the disease:

1) Subjects with high-grade glioma (grade WHO III-IV);

2) Expected survival period of 3.0 months;

3) KPS score of 70 points;

Hematology, Chemistry, and Organ Function:

1) At 1 week before randomization, the subjects' bone marrow and liver and kidney work met the following criteria:

(Hemoglobin 100g / L, neutrophils 1.5 * 109 / L, platelets 100 * 109 / L;

(1.5 times upper normal of total bilirubin, upper 1.5 times normal of aspartase aminotransferase (AST) and alanine transferase (ALT);

(Serum creatinine 1.5 times the upper limit of normal value or creatinine clearance 60 ml/min, urea nitrogen 200 mg/L;

2) Women of childbearing age must perform urine pregnancy test within 7 days before initiation and the result is negative and not in lactation. Men and women of childbearing age agreed to use reliable methods of contraception before entering the study and until six months after the end of all treatment.

2. General inclusion criteria:

1) Get the informed consent signed by the subject and his legal representative;

2) Be able to perform oral drug therapy according to the study protocol and follow-up procedures;

3) 18 years, age, 75 years.

(II) Exclusion criteria:

Any of the following were the exclusion criteria for this study:

1. Exclusion criteria related to the disease:

1) Subjects who have relapsed after previous conventional radiotherapy;

2) Those enrolled in other studies;

3) Patients with other cancers that are expected to affect subject survival;

2. General exclusion criteria:

1) Any unstable systemic disease: including active infection, uncontrolled hypertension, congestive heart failure, myocardial infarction, severe arrhythmias requiring medication, liver, kidney or metabolic diseases, neuropathy / psychosis such as Alzheimer's disease;

2) Known human immunodeficiency virus (HIV) infection;

3) Concomitant diseases that seriously endanger the safety of the subject or affect the completion of the study, and the subject's compliance with the study.

(3) Exit criteria

Subjects may terminate study treatment and evaluation at any time. The withdrawal of the subject from the study is:

1. The subject may voluntarily withdraw from the study at any time without affecting further treatment;

2. The investigator believes that there are safety problems;

3. The investigator considers that the subject has committed a serious violation of the study protocol;

4. Subject enrollment error (if the subjects did not meet the enrollment / exclusion criteria);

5. Subject was lost to follow-up;

6. Participants should also withdraw if the investigator for any reason believes it is in their best interest to withdraw from the trial.

4. Sample size estimation

According to previous reports in the literature, the study was α =0.05, β =0.2, and the expected risk value HR = 0.5. Considering the loss of the subject during the follow-up (estimated by 5%) and the enrollment of the trial group, the number of cases estimated by PASS 11 software was 152, including 76 cases in the test group and the control group.

5. Study methods and diagnostic criteria

5.1. Definition of the target area and the dose setting

Group A (Experimental arm): The target volume in the experimental arm was determined using multimodal MRI images combined with a nerve fiber bundle anatomical atlas. Gross tumor volume (GTV) was defined as resection cavity plus residual T1 contrast-enhancing tumor (if present) and categorized into 1) CTV1 comprised GTV and cytotoxic edema on image of pre- and postoperative multimodal MRI, and 2) CTV2 was expanded 1 cm only in brain white matter from CTV1, excluding adjacent brain gyrus with normal MRI signals. The margin could be modified following the margin of organs at risk. PGTV, PCTV1, and PCTV2 were determined by expanding 3 mm from GTV, CTV1, and CTV2, respectively. The prescribed Hsib-IMRT doses were 64-66, 60, and 54 Gy for PGTV, PCTV1, and PCTV2, respectively, delivered in 27 fractions, once daily and 5 times/week.

Group B (Standard arm): GTV in the standard arm was defined similarly to the experimental arm. In contrast, CTV in the standard arm were defined as GTV plus a 2 cm margin and included the cytotoxic edema area guided by multimodal MRI images according to RTOG recommendations.1 PGTV and PCTV were obtained by expanding 3 mm from GTV and CTV, respectively, with further modifications based on the margin of organs at risk. IMRT delivered 50 Gy in 25 fractions to PCTV in the first stage and delivered 10 Gy in 5 fractions to PGTV in the second stage, 2 Gy/day, 5 times/week.

5.2. Concurrent temozolomide chemotherapy and adjuvant chemotherapy

Simultemozolomide chemotherapy and adjuvant chemotherapy using the current standard treatment regimen:

A daily dose of 75mg / m^2 until the end of concurrent radiotherapy.

Four weeks after the completion of concurrent chemoradiotherapy, six cycles of adjuvant therapy were administered. Cycle 1 dose was 150mg / m^2 / day once daily for 5 days, followed by drug withdrawal for 23 days. At the beginning of cycle 2, the dose could be increased to grade 2,200mg / day for cycle 1, compared to absolute neutrophil count (ANC) 1.5 109 / L and platelet count 100 mg / m^2 / L. If the dose is not increased in cycle 2, it should not be increased in later cycles. Except for toxicity, subsequent doses were maintained at 200mg / m^2 daily.

5.3., Combined with the treatment

Subjects with seizures may be treated with antiepileptic drugs.

5.4., Concomitant medication

5.4.1. Drugs not allowed

Other anti-angiogenic and targeted therapies, including registered or investigational drugs.

5.4.2. Drugs allowed

A. Dewatering agents such as hormones and mannitol;

B. Anti-emetic therapy: conduct the corresponding anti-emetic therapy according to the specific situation;

C. White ascending drug and colony stimulating factor (G-CSF): Routine prophylactic use of G-CSF was not allowed during the study, during and after treatment due to decreased leukocytes, administer drugs according to the principle and record in medical records.

5.5. Efficacy assessment and observation indicators

5.5.1. Imaging examination

Including MRI (plain scan + enhancement), MRS, blood perfusion scan, etc

5.5.2. Main Indicators

a. Treatment effect was evaluated according to the RANO criteria:

b. Quality of life and neurocognitive function evaluation: dynamic follow-up, assessing quality of life according to the Chinese version of EORTC QLQ-C30 (V3.0), and assessing neurocognitive function according to MMSE.

5.5.3. Safety assessment

During-treatment and post-treatment adverse events were recorded.

6. Withdrawal, discontinuation, compensation, and privacy protection of the subjects

Subjects may withdraw, terminate study treatment and evaluation at any time. The details of subject withdrawal from the study include: 1. The subject may voluntarily withdraw at any time without affecting further treatment; 2. the investigator considers a safety problem; 3. the investigator considers the subject to have a serious violation of the study protocol; 4. the subject enrollment incorrectly (if the subject does not meet the enrollment / exclusion criteria); 5. the subject is lost to follow-up; 6. if the investigator considers withdrawal from the trial is in the best interest of the subject for any reason.

Our previous study found that this regimen has certain advantages in survival benefits compared with the standard treatment regimen without increasing side effects and treatment costs, so this scheme was designed for further observation and study. The current postoperative concurrent chemoradiotherapy + adjuvant chemotherapy for high-grade glioma is the standard treatment regimen in international and domestic guidelines, and it does not increase additional cost and energy to the subjects, so there is no compensation.

We will keep all your information strictly confidential and the results of this study may be reported at medical conferences and published in tech journals, but any information identifiable to you personally will not be used. You may withdraw at any time during the project without compromising your normal diagnostic treatment or your relationship with healthcare staff.

7. Data collation, input and data security management

The principal investigators of this trial were trained by GCP, fully understood the trial protocol and relevant data in advance, and strictly followed the protocol.

The case report form shall be filled in by the investigator in time to ensure the accurate content and timely summary. The case report form should be filled in with a black pen. The data and language must be accurate and clear, and should not be arbitrarily altered. If the mistakes are corrected, the horizontal lines should be drawn in the middle, and the name of the modifier and the modification time should be signed.

The completed case report form is reviewed and submitted to the data administrator for data processing (entry and management). In order to ensure the accuracy of the data, two data entry personnel are set up for data entry, comparison and verification. If there is any doubt in the case report form, the data administrator should send the question to the investigator for inquiry, and the investigator should answer and return as soon as possible. The data administrator should modify, confirm and input the data according to the investigator's answer, and send the data query information again if necessary. All of the above modifications and updates must be documented and filed.

The data of the case report form locks the database after verification and confirmation.

VIII. Statistical analysis

8.1. Data set

8.1.1. Full Analysis Data Set (FAS)

For all cases randomized and treated. Case data for the full course of treatment was not observed, the last observation was carried forward to where the trial data was missing, and the number of subjects evaluating efficacy at the endpoint was consistent with the start of the trial.

8.1.2. Conto Protocol Data Set (PPS)

Statistical analysis was performed for all the cases that met the test protocol, had good compliance, did not violate the study protocol, and completed the CRF requirements.

8.1.3. Safety Analysis Data Set (SS)

All randomized cases treated and treated with post-use safety evaluation data

constitute the safety analysis data set of the study.

8.2. Statistical analysis method

Statistical analysis was calculated using the SPSS 29 statistical analysis software.

The Kaplan-Meier method was used to evaluate disease progression-free survival and overall survival; Kaplan-Meier (log-rank) for univariate analysis and COX regression for multivariate analysis; a p-value less than or equal to 0.05 was considered statistically significant.

Measurement data are described by means and standard deviation, and use cases and percentage of counting data. First, statistical analysis was conducted on the intention-to-treat set and the compliant protocol set, and baseline analysis on the demographic characteristics of the two selected cases was conducted to investigate the equilibrium and comparability of the two groups. Then, the efficacy indicators and safety indicators of the two groups were compared.

IX. Information of the research team personnel

Name	Professional ranks and titles	Type of job	GCP training year	Research positions
Liangzhi Zhong	Physician	Tumor radiotherapy physician	In December, 2016	Patient screening and efficacy assessment
Guanghui Li	Associate chief physician	Tumor radiotherapy physician	In July, 2011	Overall research guidance
Lu Chen	Physician	Tumor radiotherapy physician	In June, 2015	Data statistics and analysis
Hongya Dai	Engineer	Radiotherapy physicist	In February, 2017	Radiotherapy plan design
Pu Zhou	Physician	Oncology	Not have	Assist in

		Physicians		chemotherapy
Shengqing Lv	Botanic physician	Neurosurgeon	Not have	Dominate the operation
Qing-rui Li	Physician	Neurosurgeon	Not have	Assist in the operation
Chun Cui	Technologist-in-charge	MRI technician	Not have	A 3D-MRI examination

方案签字页

研究者声明：

我同意严格按照本方案设计和具体规定进行临床试验，严格遵守与临床试验相关的所有的法律法规，保护患者的权利和权益。在执行该临床试验的过程中，我将严格遵守现行 GCP 和赫尔辛基宣言，并承诺整个试验过程将符合道德上的、伦理上的和科学原理上的要求。

钟良志	钟良志	2017 年 11 月 20 日
姓名（打印）	签字	日期
