# Supplementary material for: Modified Target Delineation and Moderately Hypofractionated Radiotherapy for High-Grade Glioma: A Randomized Clinical Trial
Source: JAMA Netw Open. 2025 Jul 24;8(7):e2523053. doi: 10.1001/jamanetworkopen.2025.23053 (PMC12290737; doi:10.1001/jamanetworkopen.2025.23053)
Supplement: Supplement 2. — eMethods. Target Volume Definition in Detail eTable 1. Univariate and Multivariate Analysis for Progression-Free Survival and Overall Survival eTable 2. Patterns of Recurrence [file jamanetwopen-e2523053-s002.pdf]

Supplemental Online Content

Zhong L, Zhou P, Chen L, et al. Modified target delineation and moderately hypofractionated radiotherapy for high-grade glioma: a randomized clinical trial. *JAMA Netw Open*. 2025;8(7):e2523053. doi:10.1001/jamanetworkopen.2025.23053

eMethods. Target Volume Definition in Detail
eTable 1. Univariate and Multivariate Analysis for Progression-Free Survival and Overall Survival
eTable 2. Patterns of Recurrence

This supplemental material has been provided by the authors to give readers additional information about their work.

© 2025 Zhong L et al. *JAMA Network Open.*

eMethods. Target volume definition in detail

Target volume definition of HGG patients in this study were based on merging images of simulated positioning CT and MRI. Postoperative MRI examined 2-6 weeks after surgical resection was used for target volume analysis of radiotherapy for patients with HGG.

Experimental arm:

GTV was defined as residual T1 contrast-enhancing tumor (if present) plus resection cavity of tumor lesion.

CTV1 was defined as GTV plus cytotoxic edema on image of postoperative multimodal MRI. Areas of brain edema connected to the tumor lesion only showed in T2 sequences/T2 FLAIR of preoperative MRI and disappeared in postoperative MRI would be considered as vasogenic edema. Edema area appeared in T2 sequences/T2 FLAIR of postoperative MRI and both pre and postoperative MRI would be further analyzed by DWI sequence, ADC sequence and 3-dimensions MR spectroscopy (3D-MRS). The edema with high signal on DWI sequence, low signal on ADC sequence and choline increasing on 3D-MRS would be considered as cytotoxic edema with glioma cells infiltration.

CTV2 was expanded 1 cm only in brain white matter tracts from CTV1 according to the information of nerve fiber bundle base on MR DTI sequence, excluding adjacent brain gyrus with normal MRI signals. In detail, the margin of CTV1 located at white matter would be expanded 1 cm as the boundary of CTV2 and the margin of CTV1 located at gray matter or sulci cerebri also be as the same margin of CTV2.

© 2025 Zhong L et al. *JAMA Network Open.*

Standard arm:

GTV was defined as residual T1 contrast-enhancing tumor (if present) plus resection cavity of tumor lesion.

CTV were defined as GTV plus a 2 cm margin and included the cytotoxic edema area on T2 FLAIR images based on RTOG recommendations. The margin could be modified following the margin of organs at risk. The analysis of cytotoxic edema was same as methods of experimental arm.

© 2025 Zhong L et al. *JAMA Network Open.*

eTable 1. Univariate and Multivariate Analysis for Progression-free survival and Overall Survival

Baseline Characteristics	Progression-free survival						Overall survival					
	UVA			MVA			UVA			MVA		
	HR	95%CI	*p*-value	HR	95%CI	*p*-value	HR	95%CI	*p*-value	HR	95%CI	*p* -value
**Group***				NI						NI		
	2.03	1.0-4.12	0.05				1.00	0.96-1.05	0.87			
**Gender***				NI						NI		
	0.99	0.96-1.04	0.83				0.60	0.24-1.50	0.27			
**Age**												
	2.27	1.55-3.31	＜0.001	1.75	1.19-2.57	0.004	1.79	1.19-2.68	0.005			0.39
**WHO grade**												
	2.53	1.60-3.99	＜0.001			0.05	3.2	1.8-5.5	＜0.001	2.20	1.24-	0.007
**Extent of surgery***										NI		
	0.94	0.88-1.01	0.71				1.82	0.59-5.57	0.30			
**Days to beginning RT***				NI								
	1.01	0.97-1.05	0.66				1.42	0.55-3.69	0.47			
**Adjuvant TMZ cycles**												
	0.57	0.45-0.73	＜0.001	0.63	0.50-0.80	＜0.001	0.47	0.36-0.61	＜0.001	0.50	0.39-	＜0.001
**MGMT status**												
	1.32	1.06-1.64	0.01			0.10	1.36	1.07-1.74				0.17
**IDH**												
	2.59	1.80-3.75	＜0.001	2.22	1.48-3.32	＜0.001	2.96	2.00-4.39	＜0.001	2.67	1.43-	＜0.001
**1p19q***				NI						NI		
	1.22	0.92-1.62	0.17				0.97	0.94-1.0	0.08			
**TERT_C228T***				NI						NI		
	1.06	0.85-1.31	0.62				0.98	0.96-1.01	0.23			
**TERT_C250T***				NI						NI		
	1.48	0.90-2.46	0.12				1.44	0.73-2.82	0.29			
**BRAF-V600E***				NI						NI		
	0.97	0.94-1.01	0.19				0.98	0.94-1.03	0.45			

Abbreviations: UVA: univariate analysis; HR: hazard ratio; MVA: multivariate analysis; CI: confdence interval ; RT: radiotherapy; TMZ, temozolomide; WHO, World Health Organization, MGMT, O6-methylguanine-DNA methyltransferase; IDH, isocitrate dehydrogenase; NI, not included in multivariate analysis.

* Variables violated the proportionality of hazards assumption, the HR was interpreted as weighted averages during the entire follow-up period.

.

© 2025 Zhong L et al. *JAMA Network Open.*

eTable 2. Patterns of recurrence

Experimental arm		Standard arm		*P* value
Sites of recurrence	Number of patients	Sites of recurrence	Number of patients	
Within GTV	18	Within GTV	15	.56
Within CTV1	4	Within CTV	6	>.99
Within CTV2	2			
Outside the target volume	8	Outside the target volume	15	.18
Multicentric recurrence	13	Multicentric recurrence	7	.16
Unknown	14	Unknown	12	.67

Abbreviations: GTV, gross tumor volume; CTV, clinical target volume.

© 2025 Zhong L et al. *JAMA Network Open.*
