# Supplementary material for: Modified Target Delineation and Moderately Hypofractionated Radiotherapy for High-Grade Glioma: A Randomized Clinical Trial
Source: JAMA Netw Open. 2025 Jul 24;8(7):e2523053. doi: 10.1001/jamanetworkopen.2025.23053 (PMC12290737; doi:10.1001/jamanetworkopen.2025.23053)
Supplement: Supplement 3. — Data Sharing Statement [file jamanetwopen-e2523053-s003.pdf]

Data Sharing Statement

Zhong. Modified Target Delineation and Moderately Hypofractionated Radiotherapy for High-Grade Glioma. *JAMA Netw Open*. Published July 24, 2025.
doi:10.1001/jamanetworkopen.2025.23053

Data
Additional Information: ChiCTR.org.cn, registration number: ChiCTR1800014396
Data available: Yes
Data types: Deidentified participant data
How to access data: zhongliangzhi@126.com
When available: With publication

Supporting Documents
Document types: None

Additional Information
Who can access the data: anyone requesting the data
Types of analyses: for any purpose
Mechanisms of data availability: with investigator support
